# Long-term protective potency of AAV vector-based SARS-CoV-2 prophylaxis in mice and non-human primates

**DOI:** 10.3389/fimmu.2026.1829230

**Published:** 2026-06-04

**Authors:** Ekaterina I. Ryabova, Artem A. Derkaev, Ilias B. Esmagambetov, Mikhail A. Dovgiy, Ilya V. Gordeychuk, Inna V. Shuliakova, Rosa M. Hossain, Anton A. Blinov, Anna A. Iliukhina, Daria M. Grousova, Ilya D. Zorkov, Daria V. Avdoshina, Stanislav A. Gulyaev, Vasiliy D. Apolokhov, Tatiana V. Gulyaeva, Irina A. Favorskaya, Dmitry V. Shcheblyakov, Alexander L. Gintsburg, Denis Y. Logunov

**Affiliations:** 1 N. F. Gamaleya National Research Center for Epidemiology and Microbiology, Ministry of Health of the Russian Federation, Moscow, Russia; 2 Chumakov Federal Scientific Center for Research and Development of Immune-and-Biological Products of Russian Academy of Sciences (Institute of Poliomyelitis), Moscow, Russia; 3 Institute for Translational Medicine and Biotechnology, Sechenov University, Moscow, Russia

**Keywords:** AAV vector, nanobody, long-term expression, non-human primate, passive immunization, COVID-19, SARS-CoV-2, single-domain antibody

## Abstract

**Introduction:**

Recombinant adeno-associated virus (rAAV)-mediated delivery of neutralizing antibodies is a promising strategy for rapid and durable prophylaxis against SARS-CoV-2.

**Methods:**

We evaluated the long-term expression, pharmacokinetics, immunogenicity, biodistribution, and protective efficacy of an rAAV vector of serotype DJ encoding the single-domain antibody P2C5 fused to a human Fc fragment (P2C5-Fc) in mice and common marmosets (Callithrix jacchus). Animals received a single intramuscular administration of rAAV-P2C5-Fc at a dose of 1 × 10¹³ vector genome copies per kg.

**Results:**

Rapid antibody detection in serum was observed, reaching peak concentrations (Cmax) by approximately 120 days and remaining at protective levels for >480 days in mice and up to 1,120 days in marmosets. Neutralizing titers closely paralleled serum P2C5-Fc concentrations and provided complete protection against lethal intranasal challenge with SARS-CoV-2 variants B.1.1.1 (Wuhan D614G) and BA.5 (Omicron) even at late time points (216 and 460 days post-administration in mice). Biodistribution analysis showed predominant localization of rAAV at the injection site and regional lymph nodes with minimal off-target spread. Moderate anti-AAV capsid antibody responses were detected, while anti-drug antibodies against P2C5-Fc remained undetectable in both species.

**Discussion:**

These findings demonstrate that a single intramuscular injection of rAAV-P2C5-Fc results in rapid onset, sustained expression, and long-term protective efficacy against SARS-CoV-2 variants in two preclinical models, supporting the potential of this platform for durable vectored immunoprophylaxis.

## 1 Introduction

Coronavirus disease 2019 (COVID-19), caused by severe acute respiratory syndrome coronavirus 2 (SARS-CoV-2), has resulted in >778 million confirmed cases and >7 million deaths globally since 2019 (1). To prevent severe disease, mass vaccination programs have been implemented globally. Several vaccine platforms have been developed, including inactivated, mRNA-based, and viral vector vaccines, most of which target the viral spike (S) protein (2). These vaccines have demonstrated high efficacy in reducing severe disease and mortality (3–5).

However, rapid viral evolution, the emergence of immune-evasive variants, and cohorts of immunocompromised individuals (e.g., transplant recipients, patients undergoing chemotherapy, and those with primary immunodeficiencies) limit vaccine effectiveness in certain populations (6). In addition, the development of a protective adaptive immune response following vaccination typically requires several weeks (7), which may be critical in the context of rapidly spreading viral outbreaks.

Passive immunoprophylaxis represents an alternative or complementary preventive strategy and includes the use of convalescent plasma or recombinant monoclonal antibodies (8). These approaches provide immediate protection but are limited by a relatively short duration of the protection. Therefore, alternative strategies capable of providing rapid and sustained protection are of particular interest for high-risk populations. One such strategy is passive immunization using adeno-associated virus (AAV) vectors as a delivery platform (9). Recombinant AAV (rAAV) vectors encoding genes of SARS-CoV-2-neutralizing antibodies enable sustained *in vivo* antibody production following a single administration, potentially conferring protection within days and lasting for more than one year. In some experimental settings, rAAV-based antibody delivery has been associated with long-term *in vivo* expression and sustained protective activity, including the potential for lifelong protection in a non-human primate model of HIV/SIV infection (10).

AAV is a small, non-pathogenic virus that is predominantly maintained episomally in transduced cells and is widely used in gene therapy, although rare site-specific integration events have been described for wild-type AAV (11). rAAV vectors are characterized by low immunogenicity, high stability, and the ability to mediate long-term transgene expression. Importantly, the packaging capacity of rAAV (approximately 5 kb) is sufficient to encode immunoglobulin chains, making this platform well suited for antibody gene delivery (12).

Previous studies have demonstrated the efficacy and safety of rAAV-mediated antibody delivery in models of various viral infections, including influenza viruses (13) and human immunodeficiency virus (HIV) (14). For example, Fuchs et al. showed that AAV-mediated delivery of a neutralizing antibody effectively prevented HIV infection in non-human primates, with sustained antibody production for more than six years in one animal (10). Similarly, long-term production of an antibody against Marburg virus was observed in sheep for nearly three years (15). Importantly, a first-in-human phase 1 dose-escalation study showed that intramuscular AAV8-VRC07 delivery in adults living with HIV was safe and well tolerated, with durable serum VRC07 production observed in participants during follow-up (16).

In this study, we evaluated the long-term expression, pharmacokinetics, immunogenicity, biodistribution, and protective potency of an rAAV vector of serotype DJ encoding the SARS-CoV-2-neutralizing antibody P2C5-Fc in preclinical models, including laboratory mice and common marmosets (*Callithrix jacchus*). This work extends our previous study demonstrating antibody production and protective efficacy in mice at earlier time points (17). The use of both rodent and non-human primate models enables a comprehensive assessment of the prophylactic potential of rAAV-mediated antibody delivery, accounting for interspecies differences in immune responses and rAAV transduction efficiency.

## 2 Materials and methods

### 2.1 Ethics statement

All experimental procedures involving laboratory mice were approved by the Biomedical Ethics Committee of the N.F. Gamaleya National Research Center for Epidemiology and Microbiology, Ministry of Health of the Russian Federation (Protocol No. 25; April 22, 2022).

All experimental procedures involving non-human primates were approved by the Ethics Committee of the Chumakov Federal Scientific Center for Research and Development of Immune-and-Biological Products of Russian Academy of Sciences (Institute of Poliomyelitis) (Protocol No. 31122-1; November 3, 2022).

All animal experiments were conducted and reported in accordance with ARRIVE guidelines.

### 2.2 Animals and housing

Animals were assigned to experimental groups after acclimatization in a manner ensuring comparable baseline characteristics (e.g., body weight) between groups. Investigators responsible for clinical monitoring, body weight measurements, sample collection, and survival assessment were blinded to group allocation throughout the study. Data analysis was performed using coded datasets, and group identities were revealed only after completion of primary statistical analyses.

#### 2.2.1 Mice

Laboratory mice were housed in accordance with the Guide for the Care and Use of Laboratory Animals (18, 19) and GOST (GOST - State Standard, Russian National Standard) 33216–2014 (*Guidelines for the Care and Housing of Laboratory Animals. Rules for the Care and Housing of Laboratory Rodents and Rabbits*). Animals were maintained under controlled environmental conditions at a constant temperature of 22 ± 2 °C and a relative humidity of 50%, with a 12-h light/12-h dark cycle. Mice were housed in T2-type cages, with no more than eight animals per cage. Animals had ad libitum access to a standard complete pelleted diet and drinking water.

All procedures involving laboratory mice were performed by certified veterinarians or specialists holding certification from the Russian Laboratory Animal Science Association (Rus-LASA) and trained in handling laboratory rodents. Experimental procedures were conducted in an animal facility equipped for all types of manipulations with *Mus musculus*.

Antibody expression studies were carried out using 6-week-old female BALB/c mice weighing 18–20 g (Laboratory Animal Breeding Facility, Institute of Bioorganic Chemistry, Russian Academy of Sciences, Pushchino). To evaluate protective efficacy, transgenic hemizygous K18-hACE2 mice (B6.Cg-Tg(K18-ACE2)2Prlmn/J) on a C57BL/6 background, aged 4–5 weeks, were obtained from The Jackson Laboratory (USA) and bred in accordance with official recommendations and standard husbandry practices. Mice were housed in ventilated cage systems (ISOCage P and N; Techniplast, Italy) for immunological and infectious studies conducted under biosafety level 3 (BSL-3) conditions.

#### 2.2.2 Common marmosets

Common marmosets were housed in the vivarium of the Laboratory of modeling of immunobiological processes with experimental clinic of *Callitrichidae* at the Chumakov Federal Scientific Center for Research and Development of Immune-and-Biological Products of Russian Academy of Sciences (Institute of Poliomyelitis). Animal care and husbandry complied with Sanitary Regulations (SP - Sanitary and Epidemiological Rules) 3.3686–21 (*Sanitary and Epidemiological Requirements for the Prevention of Infectious Diseases*), GOST 33218–2014 (*Guidelines for the Care and Housing of Laboratory Animals*), and Directive 2010/63/EU of the European Parliament and of the Council on the protection of animals used for scientific purposes. Detailed conditions of common marmoset housing during the experiment were described previously (20).

All procedures involving non-human primates were performed by certified veterinarians or specialists holding certification from the Federation of European Laboratory Animal Science Associations (FELASA) and trained in working with primates. Experimental procedures were conducted in a surgical facility equipped for all manipulations with common marmosets.

Animals were identified using subcutaneous radiofrequency microchips (LifeChip, Destron Fearing, USA). Body weight was measured using an electronic balance (Pioneer PA4102; Ohaus, USA).

### 2.3 Generation and production of rAAV-P2C5-Fc

The single-domain antibody P2C5 modified with a human Fc fragment (P2C5-Fc) was generated as previously described (21). The rAAV-P2C5-Fc vector construct was produced using a three-plasmid AAV-DJ packaging system (Cell Biolabs, USA) by transient transfection of HEK293 cells (obtained from the cell collection of the N.F. Gamaleya National Research Center for Epidemiology and Microbiology, Ministry of Health of the Russian Federation), followed by cell cultivation and affinity purification using AVB Sepharose resin (Cytiva Life Sciences, USA). The complete production cycle of the vector construct has been described in earlier publications (22, 23).

### 2.4 Vector Administration and Sample Collection

For the experiments described in this study, experimental groups of female mice received intramuscular (i.m.) injections of the rAAV-P2C5-Fc vector into the femoral muscle of the right or left hind limb (0.1 mL). Control animals received an equivalent volume of buffer administered via the same route. Serum preparation and analysis, assessment of P2C5-Fc expression, immunogenicity, and neutralizing antibody (NAb) activity at various time points were performed as previously described (17).

To assess expression, pharmacokinetics, and neutralizing antibody activity of the modified single-domain antibody P2C5-Fc with neutralizing activity against multiple SARS-CoV-2 variants, experimental groups of marmosets received i.m. injections of purified rAAV-P2C5-Fc (0.5 mL) into the femoral muscle of the right or left hind limb. Control animals received an equivalent volume of buffer administered via the same route.

Blood samples were collected immediately prior to vector administration and at various time points thereafter from animals in both groups by femoral vein puncture using 2.5-mL three-component syringes fitted with 27G needles. The maximum single blood collection volume did not exceed 3 mL. Blood samples were transferred to tubes, gently mixed, and incubated at 37 °C for 1 h. Serum was separated by centrifugation (5810R; Eppendorf, Germany) at 600 × g for 10 min, aliquoted, and stored at -80 °C for subsequent analysis of antibody expression, neutralizing activity, and immunogenicity.

### 2.5 Intranasal SARS-CoV-2 challenge

To evaluate protective efficacy against SARS-CoV-2 infection, mice were intranasally (i.n.) challenged with different viral variants at a dose of 1 × 10^5^ TCID_50_ per animal, as previously described (17). SARS-CoV-2 variants B.1.1.1 (PMVL-1; GISAID EPI_ISL_421275; Wuhan D614G) and BA.5 (Omicron) (hCoV-19/Russia/SPE-RII-25357S/2022), originally isolated from nasopharyngeal swabs, were obtained from the State Collection of Viruses of the N.F. Gamaleya National Research Center and were used both for infection experiments and neutralizing antibody assays.

### 2.6 Determination of serum P2C5-Fc concentrations

Serum concentrations of the P2C5-Fc antibody in mice were determined by indirect enzyme-linked immunosorbent assay (ELISA) using the receptor-binding domain (RBD) protein of the SARS-CoV-2 B.1.1.1 (PMVL-1; Wuhan D614G) variant as the coating antigen (100 ng/well in carbonate-bicarbonate buffer, pH 9.6, overnight at 4 °C). After blocking with 5% non-fat milk in PBS-T for 1 h at 37 °C, serial dilutions of serum samples were added and incubated for 1 h at 37 °C. Horseradish peroxidase (HRP)-conjugated Human IgG Whole Antibody (Cytiva, USA) was used as the secondary antibody (1:2500 dilution, 1 h at 37 °C). A calibration curve was generated using purified P2C5-Fc standards of known concentrations. The assay sensitivity (limit of detection) was approximately 2 ng/mL, with intra- and inter-assay coefficients of variation 15-20%.

### 2.7 Assessment of serum P2C5-Fc levels, neutralizing activity, immunogenicity, and pharmacokinetics

NAb activity was assessed by microneutralization assay using live SARS-CoV-2 B.1.1.1 (PMVL-1; Wuhan D614G) strain as previously described (17). Briefly, heat-inactivated serum samples were serially diluted two-fold starting from 1:10 in complete DMEM supplemented with 2% heat-inactivated fetal bovine serum and incubated with 100 TCID_50_ of SARS-CoV-2 for 1 h at 37 °C. The mixture was added to Vero E6 cell monolayers in 96-well plates and incubated for 96–120 h at 37 °C with 5% CO_2_. The cytopathic effect (CPE) was assessed visually, and the endpoint neutralizing titer was defined as the highest dilution that completely inhibited CPE in at least two of three replicate wells.

Immunogenicity against the AAV-DJ capsid and the expressed P2C5-Fc was evaluated by indirect ELISA as described (17). For detection of anti-AAV capsid antibodies, plates were coated with purified rAAV-P2C5-Fc vector particles (or empty AAV-DJ capsid). For detection of anti-P2C5-Fc (anti-drug) antibodies, plates were coated with purified recombinant P2C5-Fc protein. Serial dilutions of serum samples were added and incubated for 1 h at 37 °C, followed by HRP-conjugated species-specific secondary antibodies (anti-mouse or anti-marmoset IgG). Titers were expressed as the reciprocal of the highest dilution giving an optical density value above the cut-off.

Pharmacokinetic parameters were calculated using non-compartmental analysis (NCA). AUC_0_–t was determined by the linear trapezoidal method. Terminal elimination rate constant (λz) was estimated by log-linear regression of the post-peak declining phase. AUC_0_–∞, AUMC_0_–∞ and mean residence time (MRT) were calculated using standard extrapolation methods. Due to continuous antibody expression from the AAV vector, all elimination parameters are reported as apparent values.

### 2.8 rAAV biodistribution

To assess rAAV biodistribution, experimental mice were euthanized and subjected to necropsy for collection of major organs. Tissues were homogenized using a FastPrep-24 homogenizer (MP Biomedicals, USA) with 1.4-mm ceramic beads, followed by genomic DNA extraction using the Wizard Genomic DNA Purification Kit (Promega, USA). Vector genome copy numbers were quantified by real-time PCR using the AAVpro Titration Kit (for Real-Time PCR), Ver. 2 (Takara Bio, Japan) and a CFX96 real-time PCR system (Bio-Rad, USA).

### 2.9 Statistical analysis

Data analysis was performed using Microsoft Office Excel 2010, GraphPad Prism version 9.0 (USA), and ELISA Master software (AlkorBio, Russia). Intergroup comparisons of antibody titers, animal survival, and other quantitative parameters were performed using the Mann-Whitney U test and the Gehan-Breslow-Wilcoxon test, with a significance level of 0.05. Median survival was calculated using Kaplan-Meier survival analysis. Statistical significance of differences was additionally assessed using Student’s t-test and the log-rank (Mantel-Cox) test.

Group sizes were determined based on effect sizes observed in preliminary experiments and prior experience with similar *in vivo* models. The selected numbers of animals were considered sufficient to detect biologically relevant differences between treated and control groups using appropriate statistical methods.

For survival experiments, group sizes were chosen to allow reliable detection of substantial differences in survival probability using log-rank testing. Final group sizes were selected to ensure statistical validity while minimizing animal use in accordance with the 3R principles.

## 3 Results

As the present study represents a continuation of previously published work, the generation and characterization of the rAAV-P2C5-Fc vector were identical to those described earlier (17).

### 3.1 rAAV-P2C5-Fc confers durable protection against SARS-CoV-2 variants for at least 460 days in mice with predominant localization at the injection site

**Figure 1:**
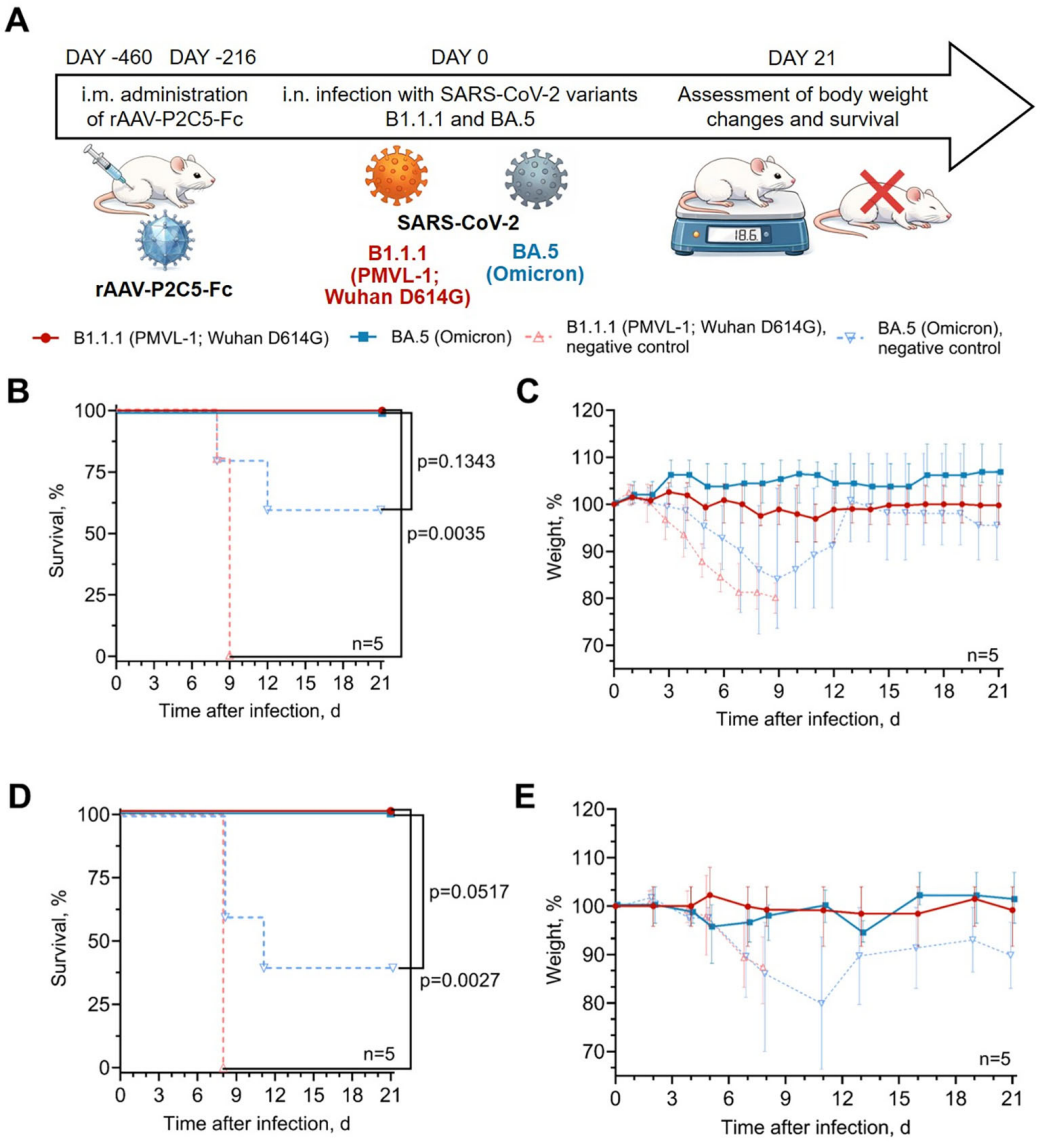
Analysis of survival and body-weight changes of mice over 21 days following i.n. challenge with SARS-CoV-2 variants B.1.1.1 (PMVL-1; Wuhan D614G) and BA.5 **(Omicron)** (1×10^5^ TCID_50_), at various time points after i.m. administration of rAAV-P2C5-Fc at a 1×10^13^ gc/kg. **(A)** Study design. **(B)** Survival of mice infected 216 days post-treatment. **(C)** Body-weight change (%) for mice challenged 216 days post-treatment. **(D)** Survival of mice infected 460 days post-treatment. **(E)** Body-weight change (%) for mice challenged 460 days post-treatment.

In our previous study, we demonstrated protection of mice against SARS-CoV-2 infection from day 1 to day 140 post-administration (17). To evaluate the duration and protective efficacy of rAAV-P2C5-Fc, groups of five K18-hACE2 transgenic mice received a single i.m. dose of rAAV-P2C5-Fc at a 1 × 10^13^ gc/kg (2 × 10^11^ gc per animal) and were i.n. challenged with SARS-CoV-2 variants B.1.1.1 (PMVL-1; Wuhan D614G) and BA.5 (Omicron) at 1 × 10^5^ TCID_50_ per animal at 216 and 460 days post-administration (**Figure 1A**). Control groups (n = 5) received buffer instead of rAAV-P2C5-Fc and were challenged at the same time points. Animals were monitored for 21 days following infection.

At 216 days post-administration, treated animals showed 100% survival after challenge with B.1.1.1 (PMVL-1; Wuhan D614G) and 100% survival after challenge with BA.5 (Omicron) (**Figure 1B**). Corresponding control groups exhibited 0% and 60% survival, respectively. We note that the BA.5 challenge at this late time point induced only moderate lethality in control animals (60% survival), which is consistent with the reduced pathogenicity of Omicron BA.5 and other Omicron subvariants in the K18-hACE2 mouse model compared with ancestral strains (24, 25). Nevertheless, animals that received rAAV-P2C5-Fc were fully protected (100% survival with no significant body-weight loss). Treated mice did not experience significant body-weight loss, in contrast to the control groups (**Figure 1C**).

A similar protective effect was observed following challenge at 460 days post-administration: treated groups showed 100% survival, whereas the control group challenged with B.1.1.1 (PMVL-1; Wuhan D614G) exhibited complete mortality (0% survival) and the control group challenged with BA.5 (Omicron) showed 40% survival (i.e., 60% mortality) (**Figure 1D**). No significant body-weight changes were observed throughout the observation period (**Figure 1E**).

**Figure 2:**
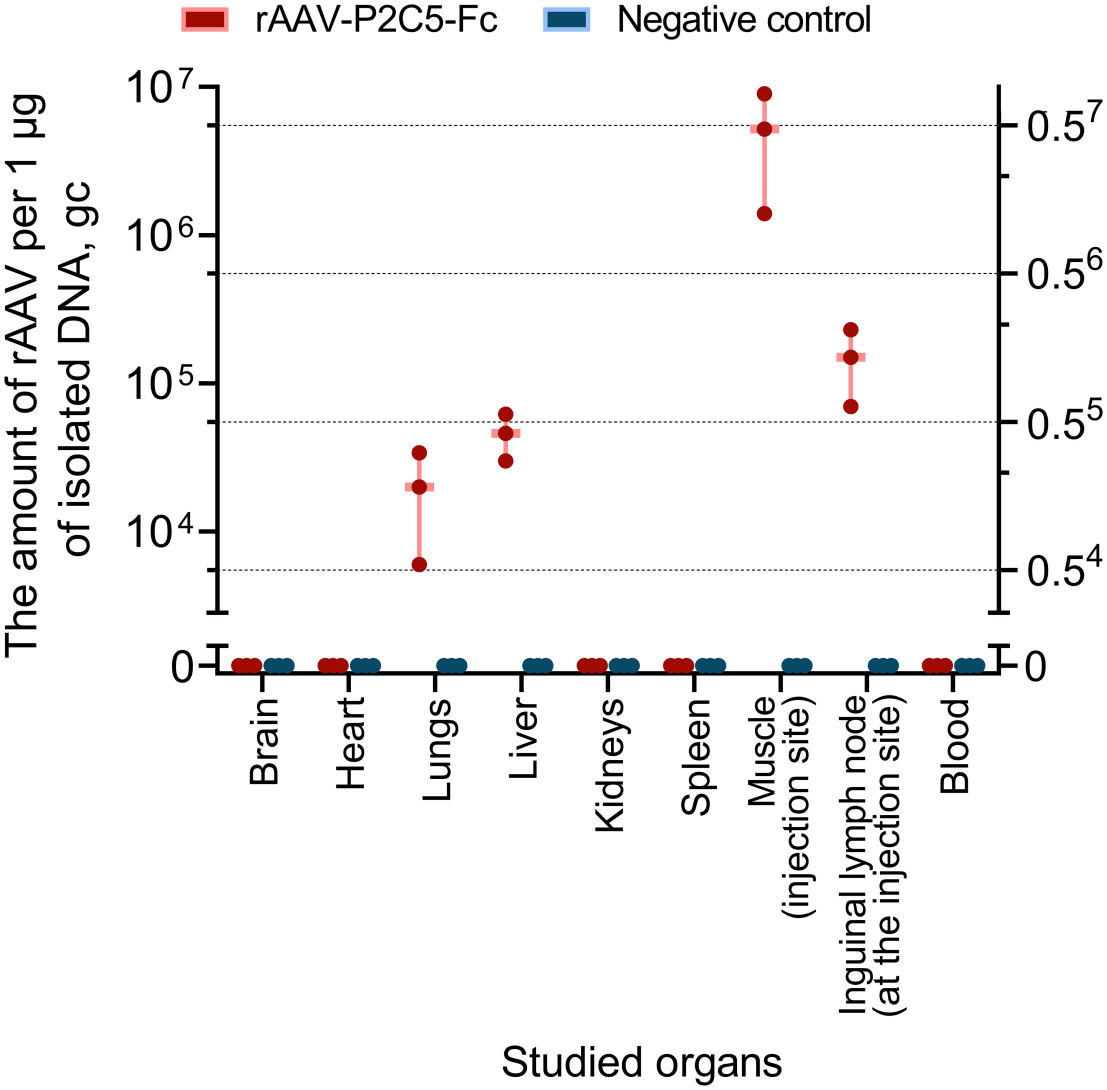
Analysis of rAAV vector genome copy numbers (per 1 µg of extracted DNA) in major organs of mice at 7 days after i.m. administration of rAAV-P2C5-Fc at a dose of a 1 × 10^13^ gc/kg.

To investigate the tissue distribution of P2C5-Fc expression, the biodistribution of rAAV-P2C5-Fc was analyzed in BALB/c mice by quantitative real-time PCR. Following i.m. administration of rAAV-P2C5-Fc at a working dose of a 1 × 10^13^ gc/kg to a group of three animals, major organs were analyzed for the presence of rAAV DNA at 7 days post-administration. rAAV genomes were detected at the injection site (femoral muscle; 5.2 × 10^6^ gc/µg), in the inguinal (draining) lymph node (1.5 × 10^5^ gc/µg), in the liver (1.5 × 10^5^ gc/µg), and in the lungs (2.0 × 10^4^ gc/µg) (**Figure 2**). No ITR sequences were detected in brain, heart, kidneys, spleen, or blood.

Thus, the carried-out studies yielded data demonstrating prolonged and highly effective protective activity of rAAV-P2C5-Fc in mouse models against multiple SARS-CoV-2 variants.

### 3.2 rAAV-P2C5-Fc sustains P2C5-Fc production and neutralizing activity for at least 480 days in mice with anti-capsid but no anti-P2C5-Fc responses

To confirm the sustained *in vivo* expression of the P2C5-Fc antibody and to establish the pharmacokinetic basis for the long-term protective efficacy observed in the SARS-CoV-2 challenge studies, we evaluated the kinetics of serum P2C5-Fc concentrations and virus-neutralizing activity in BALB/c mice over an extended period following a single intramuscular administration of rAAV-P2C5-Fc.

**Figure 3:**
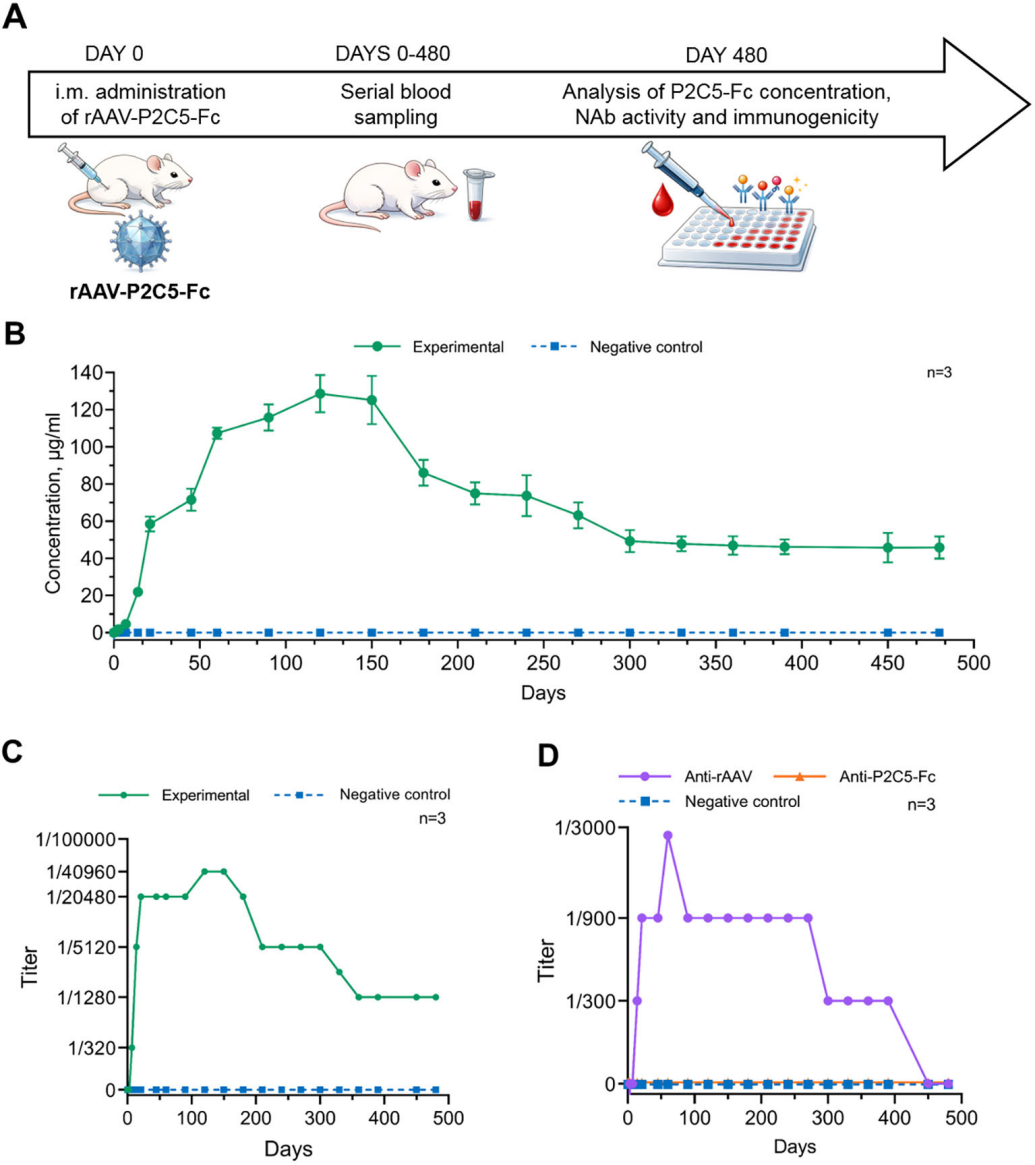
Analysis of serum P2C5-Fc concentration, neutralizing activity, and immunogenicity in mice following i.m. administration of rAAV-P2C5-Fc. **(A)** Study design. **(B)** Serum levels of the P2C5-Fc antibody following i.m. administration of rAAV-P2C5-Fc at a dose of a 1.0 × 10^13^ gc/kg in mice. **(C)** NAb activity titers against SARS-CoV-2. **(D)** Antibody titers against AAV-DJ capsid proteins and the expressed P2C5-Fc antibody.

Following i.m. administration of purified and characterized rAAV-P2C5-Fc at the optimal protective dose of a 1.0 × 10^13^ gc/kg, serum levels of the protective antibody P2C5-Fc were analyzed in groups of three BALB/c mice at various time points (**Figure 3A**). Groups of three mice receiving buffer alone via the same route served as negative controls. Antibody concentrations were quantified by ELISA using the SARS-CoV-2 RBD as the coating antigen.

P2C5-Fc was detectable throughout the entire observation period, with sustained expression for at least 480 days following rAAV-P2C5-Fc administration (**Figure 3B**). Further follow-up was limited by the advanced age of the animals. Serum antibody concentrations peaked at 128.6 µg/mL at 120 days post-injection and declined thereafter, remaining at approximately 49.2-45.8 µg/mL between 300 and 480 days. Serum concentration–time data were subjected to non-compartmental pharmacokinetic analysis (NCA) using the linear trapezoidal rule for AUC calculation and log-linear regression for the terminal elimination phase. The derived pharmacokinetic parameters for mice are summarized in **Table 1**.

**Table 1:** Main pharmacokinetic parameters for rAAV-P2C5-Fc in the experimental animals.

Parameter	Description	Units	Value	
			Mice	Marmosets
T_1_/_2_	Half-life	days	228.0	94.93
T_max_	Time to C_max_	days	120	168
C_max_	Maximum concentration	µg/mL	128.6	209.8
AUC (0-t)	Area under the concentration-time curve	µg × day/mL	34057.4	119403.13
AUC (0-∞)	Area under the concentration-time curve extrapolated to infinity	µg × day/mL	49122.7	131555.73
AUMC (0-∞)	Area under the first moment curve	µg × day^2^/mL	1.92 × 10^7^	7.51 × 10^7^
MRT	Mean residence time	days	391.3	571.2
K_el_	Elimination rate constant	days^-1^	0.00304	0.0073014
R^2^	Coefficient of determination (goodness of fit)	–	0.8294	0.9844

The kinetics of virus-neutralizing antibody activity closely correlated with serum P2C5-Fc concentrations. Neutralizing antibody titers, as determined by ELISA, reached a maximum of 1:40960 at 120 days after vector administration and decreased to 1:5120-1:1280 at 300–480 days, respectively (**Figure 3C**).

To assess the immunogenicity of rAAV-P2C5-Fc, mouse sera were analyzed by ELISA for the presence of antibodies against the AAV-DJ capsid proteins (anti-rAAV) and against the expressed P2C5-Fc antibody (anti-P2C5-Fc); rAAV-P2C5-Fc and purified P2C5-Fc were used as coating antigens for the detection of anti-rAAV and anti-P2C5-Fc antibodies, respectively. Anti-rAAV antibodies were detected, reaching a peak titer of 1:2700 at 60 days after administration of rAAV-P2C5-Fc, followed by a decline to 1:900 (**Figure 3D**). In contrast, anti-P2C5-Fc antibody levels were below the limit of detection and did not differ significantly from those in the negative control group.

### 3.3 rAAV-P2C5-Fc sustains serum P2C5-Fc levels for at least 1120 days in common marmosets with a prolonged plateau phase and minimal anti-P2C5-Fc immunogenicity

Having demonstrated long-term protective efficacy and sustained antibody expression in mouse models, we next evaluated the pharmacokinetics, durability of expression and immunogenicity of rAAV-P2C5-Fc in common marmosets (*Callithrix jacchus*) — a non-human primate species phylogenetically closer to humans and widely used for translational studies of AAV-based gene therapy. Experimental and control groups of marmosets were formed randomly; the animals’ characteristics are presented in **Table 2**. The experimental group received an i.m. injection of rAAV-P2C5-Fc at a dose of a 1 × 10^13^ gc/kg, while control animals received an equivalent volume of buffer via the same route. Blood samples were collected at various time points and sera were prepared for subsequent analyses. Using a solid-phase ELISA with the SARS-CoV-2 receptor-binding domain (RBD) — following the same protocol applied to mouse sera — we determined the time course of serum P2C5-Fc concentrations up to 1120 days post-administration and calculated the main pharmacokinetic parameters (**Table 1**).

**Table 2:** Characteristics of common marmosets used in the study .

Animal ID	Group	Sex	Age, months	Weight, g
1	Experimental	♀	20	400
2	Experimental	♂	19	370
3	Experimental	♂	16	380
4	Control	♂	22	430
5	Control	♂	27	460
6	Control	♂	21	430

In mice, the maximum serum concentration (C_max_) of 128.6 µg/mL was reached at day 120 (T_max_). The observed exposure (AUC (0-t)) was 34057 µg × day/mL. Terminal slope estimation using data from day 120 onward yielded an apparent elimination rate constant λz of 0.00304 day^-1^, corresponding to an apparent half-life of 228 days. Extrapolation beyond the last sampling point accounted for 30.7% of AUC (0-∞).

In marmosets, serum antibody concentrations increased gradually, reaching peak levels between days 147-168, followed by a prolonged plateau phase. Non-compartmental analysis showed sustained systemic exposure (AUC (0-t) = 119403 µg × day/mL). Estimation of the terminal slope was highly sensitive to time-window selection: post-peak decline analysis yielded an apparent half-life of approximately ~95 days, whereas inclusion of late plateau-phase data resulted in near-zero slopes and unrealistically long half-life estimates, reflecting steady-state conditions rather than intrinsic elimination.

Thus, while both species demonstrated long-term antibody exposure following AAV administration, the shape of the concentration-time profiles and the reliability of elimination-related parameters differed substantially between mice and marmosets.

**Figure 4:**
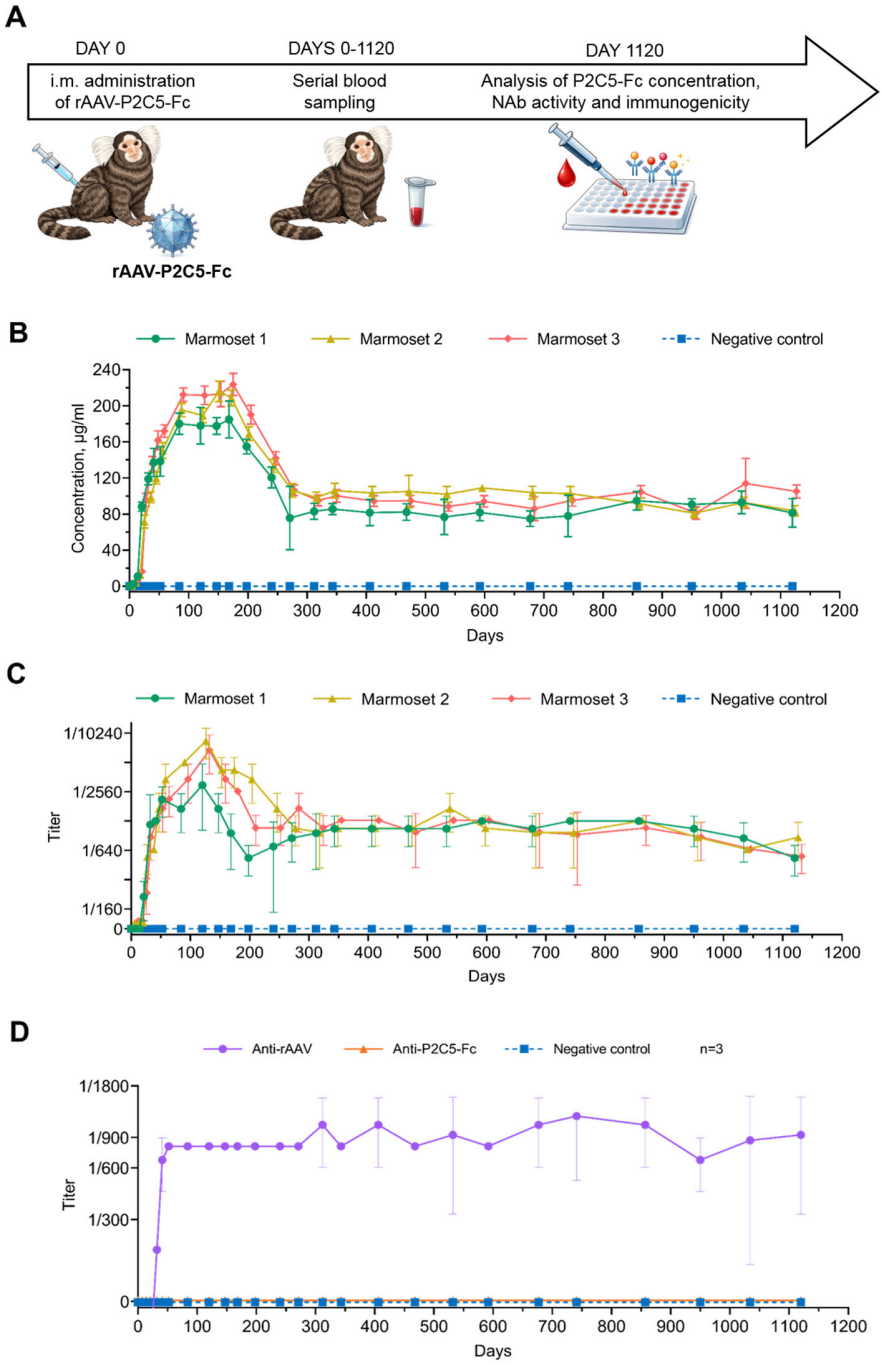
Analysis of serum P2C5-Fc concentration, neutralizing activity, and immunogenicity in marmosets following i.m. administration of rAAV-P2C5-Fc. **(A)** Study design. **(B)** Serum concentrations of the P2C5-Fc antibody in marmosets following i.m. administration of rAAV-P2C5-Fc at a dose of a 1.0 × 10^13^ gc/kg. **(C)** NAb activity titers against SARS-CoV-2. **(D)** Antibody titers against rAAV capsid proteins and the expressed P2C5-Fc antibody.

The presence of the protective antibody was demonstrated throughout the entire duration of the study (**Figure 4A**). Following administration of the investigational product, a rapid increase in serum P2C5-Fc concentration was observed, reaching a peak level of 209.8 µg/mL at 168 days after rAAV-P2C5-Fc administration (**Figure 4B**). Over time, antibody concentrations gradually declined, reaching levels of ~94.8-88.7 µg/mL at 271–1120 days post-administration, respectively.

Neutralizing antibody activity closely correlated with changes in P2C5-Fc serum concentrations. Neutralizing antibody titers reached 1:10240 at 120 days after rAAV-P2C5-Fc administration and subsequently declined to 1:5120-1:640 at 168–1120 days, respectively (**Figure 4C**).

To assess the immunogenicity of rAAV-P2C5-Fc, marmoset sera were analyzed by ELISA for the presence of antibodies against rAAV capsid proteins (anti-rAAV) and against the expressed P2C5-Fc antibody (anti-P2C5-Fc), using rAAV-P2C5-Fc and purified P2C5-Fc antibody as coating antigens, respectively. Anti-rAAV antibodies were detected, reaching a peak titer of 1:800 at 52 days after rAAV-P2C5-Fc administration, after which the titer remained at this level throughout the 1120-day observation period (**Figure 4D**). In contrast, anti-P2C5-Fc antibody levels remained low (below the limit of detection) for the entire duration of the experiment and did not differ significantly from those observed in the control group.

## 4 Discussion

In this study, we demonstrated long-term *in vivo* expression and sustained protective efficacy of an rAAV vector encoding the neutralizing single-domain antibody P2C5-Fc in two complementary preclinical models. A single i.m. administration of rAAV-P2C5-Fc at a 1 × 10^13^ gc/kg resulted in persistent serum antibody levels and durable neutralizing activity that were maintained for many months: in mice P2C5-Fc was detectable for at least 480 days (follow-up beyond this point was precluded by age-related mortality), with a peak serum concentration of ≈128.6 µg/mL and a peak neutralizing antibody titer of up to 1:40960, whereas in common marmosets antibody expression persisted to 1,120 days with a peak C_max_ ≈209.8 µg/mL and neutralizing titers reaching 1:10240. In mice, a strong positive correlation was observed between binding antibody levels (ELISA) and neutralizing activity. In contrast, in marmosets this correlation was weaker and showed greater dispersion, particularly at later time points (>168 days). This apparent discrepancy does not indicate reduced functionality of the expressed antibody but rather reflects fundamental differences between the assays and species-specific factors. The microneutralization assay measures functional neutralizing activity of P2C5-Fc, whereas the ELISA quantifies total antigen-binding antibody levels (26, 27). These differences, likely account for the weaker correlation observed at later time points. More importantly, the marmoset data may appear atypical compared with many previous AAV-mediated monoclonal antibody studies because the majority of those studies delivered traditional full-length IgG molecules (28), whereas we expressed a single-domain nanobody-Fc (VHH-Fc) fusion protein. The smaller size, distinct structure, and different post-translational modification profile (e.g., glycosylation patterns produced by primate muscle cells) of the VHH-Fc format can result in species-dependent differences in serum matrix effects, epitope accessibility, and the quantitative relationship between total antigen-binding capacity and functional neutralizing potency. Such format- and species-dependent variation in the correlation between binding and neutralizing titers has been noted in other vectored antibody studies (26, 27).

Taken together, although measured neutralizing titers were lower in marmosets, this finding should be interpreted in the context of species-specific differences in antibody quality and assay characteristics rather than as evidence of reduced protective potential. These observations underscore the value of using both binding and functional assays when evaluating vectored immunoprophylaxis, especially with novel antibody formats such as nanobody-Fc fusions.

Direct comparison of pharmacokinetic parameters between mice and marmosets highlights species-specific differences in expression kinetics and steady-state behavior. In mice, a relatively well-defined post-peak decline allowed estimation of an apparent half-life from day 120 onward. In contrast, marmosets exhibited a broader peak followed by a prolonged plateau, causing automatic terminal-phase selection to yield artificially long half-life and MRT values. These findings illustrate a key limitation of non-compartmental analysis for AAV-mediated antibody delivery, where continuous transgene expression violates the assumption of a single-dose input. Consequently, exposure metrics such as C_max_ and AUC (0-t) are the most robust for cross-species comparison, whereas elimination-related parameters should be interpreted qualitatively.

The K18-hACE2 mouse model, while highly stringent and associated with severe disease and mortality following SARS-CoV-2 challenge, is particularly well suited for evaluating functional protection and minimal protective antibody thresholds. Although this model may overestimate disease severity compared with typical human infection, the consistent survival benefit and absence of significant body weight loss observed even at late time points strongly indicate that sustained low-to-moderate circulating levels of P2C5-Fc are sufficient to confer effective prophylactic protection. Therefore, the mouse data primarily support conclusions regarding durability of expression and functional efficacy rather than direct quantitative extrapolation of protective titers to humans.

Compared with other AAV-based approaches specifically developed for SARS-CoV-2 prophylaxis, our data demonstrate comparable or superior durability and efficacy. For example, a recent study employing hepatotropic (AAV8) and myotropic (AAVMYO) vectors for delivery of a SARS-CoV-2 neutralizing antibody (TRES6) in mice, administered at a dose of a 1 × 10^13^ gc/kg, reported protection from SARS-CoV-2 infection in K18-hACE2 mice at day 14 post-administration and sustained expression for up to 52 weeks, with peak antibody levels of approximately 110 µg/mL and 511 µg/mL at week 24 following AAV8 and AAVMYO administration, respectively, followed by maintenance of concentrations above 40 µg/mL and 100 µg/mL, respectively, for one year (29). In contrast to i.n. AAV delivery approaches using ACE2 decoys or CRISPR-Cas13d systems that provide prophylactic protection against SARS-CoV-2 variants in mice and hamsters (30, 31), our i.m. approach using a nanobody-Fc fusion provides systemic protection with minimal anti-drug immunogenicity, thereby addressing limitations associated with mucosal targeting. Collectively, the present work confirms the generalizability of the AAV-based antibody delivery platform beyond HIV and influenza models (13, 15) and underscores its applicability to emerging respiratory viruses with demonstrated efficacy against current SARS-CoV-2 variants of concern.

AAV-based prophylaxis raises two central immunological questions: host responses to the vector capsid and immune recognition of the expressed therapeutic antibody. In our experiments with the rAAV-P2C5-Fc (AAV-DJ) vector, anti-capsid responses were measurable but moderate – the anti-AAV titers peaked (mouse: 1:2700 at day 60; marmoset: 1:800 at day 52) and subsequently declined to a stabilized moderate level – while anti-P2C5-Fc responses remained below the limit of detection in both species throughout the observation period and did not differ from controls, indicating low immunogenicity of the Fc-modified single-domain antibody in these models. Importantly, despite the use of fully immunocompetent wild-type BALB/c mice, we did not detect anti-drug antibodies (ADA) against the P2C5-Fc fusion protein throughout the 480-day observation period (**Figure 3D**). The minimal immunogenicity observed here is most likely attributable to the intrinsic low-immunogenicity profile of the camelid single-domain antibody (VHH/nanobody) scaffold (32) and to the sustained high-level transgene expression achieved via intramuscular AAV delivery, which is known to promote antigen-specific immune tolerance in immunocompetent mice (33). In support of this, a related study using a similar AAV-delivered human Fc–fused VHH construct also showed only a minimal anti-VHH-Fc signal (23). Taken together, the VHH-Fc format combined with continuous local intramuscular expression appears to enable durable and immunologically well-tolerated antibody production even in fully immunocompetent animals.

Biodistribution analysis at day 7 post-administration confirmed predominant localization of rAAV genomes at the injection site (femoral muscle) and draining inguinal lymph node, with only minimal detection in liver and lungs and no detection in brain, heart, kidneys, spleen, or blood (**Figure 2**). Although vector genome quantification was performed only at this early time point, the sustained functional expression and protective efficacy of P2C5-Fc for >480 days in mice and up to 1,120 days in common marmosets, together with the absence of any toxicity or anti-drug antibody responses, strongly support the well-established long-term episomal persistence of rAAV vectors primarily in post-mitotic skeletal muscle cells with minimal redistribution to non-target tissues after intramuscular delivery (12, 34). We also indirectly confirmed localized persistence using the same rAAV platform by analyzing luciferase reporter activity at various time points, which remained predominantly at the injection site (17). Together, these data support a favorable immunogenicity and biodistribution profile in both species, although important caveats regarding repeat dosing, preexisting anti-AAV immunity and clinical translation remain (12, 35).

Compared with conventional vaccines and passive monoclonal antibody infusion, rAAV-mediated antibody delivery combines several practical advantages. Unlike vaccination, vectored antibody delivery can provide more rapid functional protection without the need for the host to develop an adaptive immune response – an important benefit for immunocompromised individuals and during rapidly evolving outbreaks (6, 7). Compared with repeated recombinant antibody infusions, a single rAAV dose can sustain protective antibody concentrations for months to years, reducing logistical and economic burdens and improving compliance (8). Local i.m. delivery concentrates transgene expression in a predictable site and facilitates manufacturing and dosing. Our data – rapid achievement of high serum concentrations and durable NAb titers that translate into protection against heterologous challenge – illustrate these practical advantages. In challenge experiments, rAAV-treated animals exhibited minimal body weight loss and high survival rates even at late time points, suggesting that sustained low-to-moderate antibody levels (e.g., 45-100 µg/mL) are sufficient for prophylactic protection. Observed protection against B.1.1.1 (PMVL-1; Wuhan D614G) and BA.5 (Omicron) underscores the broad neutralizing potential of P2C5-Fc and contrasts with many spike-based vaccines and monoclonals that have lost potency against emerging variants (6).

We acknowledge that the *in vivo* protective efficacy was evaluated only against the ancestral B.1.1.1 and BA.5 variants. While these challenges provide strong evidence of long-term durability against both early and antigenically distinct Omicron lineages, more recently emerged immune-evasive variants (e.g., XBB and JN.1/KP sublineages) were not included. The P2C5 nanobody was selected based on its previously demonstrated broad neutralizing activity against multiple VOCs, including Omicron BA.1 (21). Structural analyses suggest that certain mutations (e.g., F490S in XBB lineages) may reduce P2C5 binding and neutralization (36). Nevertheless, the present work establishes the rAAV-P2C5-Fc platform as a valuable preclinical model for assessing the long-term protective potential of vectored immunoprophylaxis. Future studies will therefore evaluate the efficacy of sustained rAAV-P2C5-Fc expression against currently circulating SARS-CoV-2 lineages to further support clinical translation.

Although survival and body weight change were used as the primary endpoints in these long-term challenge experiments, we previously demonstrated using the identical rAAV-P2C5-Fc vector and K18-hACE2 mouse model that protection is directly associated with a significant reduction in SARS-CoV-2 viral load in the lungs, as measured by quantitative RT-PCR (17). These data confirm that the durable clinical protection observed in the current study results from effective suppression of viral replication mediated by sustained circulating levels of P2C5-Fc.

Despite the encouraging preclinical results, several translational challenges must be considered. First, preexisting humoral immunity to AAV in humans may limit transduction and efficacy; seroprevalence varies by serotype and geography and may necessitate capsid engineering, selection of low-seroprevalence serotypes, or immune-modulatory regimens (37, 38). Pre-existing neutralizing antibodies against AAV capsids represent a major translational barrier for AAV-based gene therapies in humans. Seroprevalence of anti-AAV neutralizing antibodies ranges from 30% to over 70% depending on serotype and geographic region, and even low titers can potently inhibit vector transduction, markedly reduce transgene expression levels, and preclude effective re-dosing (37, 38). Although the present study was conducted in immunologically naïve mice and common marmosets – where only moderate *de novo* anti-capsid responses developed without compromising sustained P2C5-Fc expression for up to 1,120 days – this pre-existing immunity will require careful consideration in clinical development. Several promising strategies are currently being pursued to mitigate this challenge, including the use of engineered or naturally low-seroprevalence AAV capsids, transient immunosuppression, plasmapheresis, and enzymatic IgG depletion (e.g., imlifidase/IdeS) (39). Systematic evaluation of these approaches, together with pre-treatment patient screening, will be critical to broaden patient eligibility and ensure robust therapeutic efficacy upon clinical translation.

Importantly, the translational potential of intramuscular AAV-mediated antibody delivery has already been demonstrated in a first-in-human phase 1 dose-escalation study of AAV8-mediated delivery of the anti-HIV monoclonal antibody VRC07. This trial showed that the approach was safe and well tolerated, with durable serum VRC07 production observed during follow-up (16). Second, species differences in AAV tropism and immune responses mean that durability and expression levels observed in mice and marmosets may not fully predict human outcomes; protective neutralizing thresholds for humans remain to be defined. Third, anti-drug antibodies (ADA) against the expressed antibody could arise and reduce efficacy over time; although ADA were negligible in our models, human immune repertoires differ and ADA monitoring will be essential in clinical development. Finally, manufacturing and dose-scaling of clinical-grade AAV vectors for prophylactic use represent substantial logistical and economic hurdles.

In the present study, murine models were used to establish direct proof-of-protection against lethal SARS-CoV-2 challenge, whereas marmosets were employed to characterize long-term expression, pharmacokinetics, and immunogenicity of the vectored antibody in a primate system. This complementary approach allows functional efficacy to be demonstrated in a highly sensitive challenge model, while leveraging the non-human primate data to gain insight into durability, antibody quality, and host-vector interactions that are more relevant for clinical translation.

Taken together, the complementary results obtained in mice and in a non-human primate model highlight distinct yet clinically relevant aspects of long-term, AAV-mediated antibody expression and underscore the value of multi-species evaluation for translational development. While murine studies enable efficient assessment of expression kinetics, functional activity, and protective efficacy, non-human primate data provide critical insight into antibody quality, durability, and species-dependent factors that are likely to be more predictive of human outcomes. Viewed in this context, the combined datasets inform several rational next steps for further development, including optimization of capsid and expression cassette design to improve transduction of human skeletal muscle (e.g., promoter selection, signal peptide choice, Fc engineering, and codon optimization), evaluation of strategies to mitigate anti-capsid immunity (such as transient immunosuppression, plasmapheresis, or engineered capsids), and assessment of vectored antibody delivery against an expanded panel of SARS-CoV-2 variants. In addition, combination approaches-such as vectored delivery of multispecific antibodies or integration of rAAV-based prophylaxis with conventional vaccination-may further enhance the breadth, robustness, and durability of protective immunity.

## 5 Conclusion

In summary, our preclinical data show that rAAV-mediated delivery of P2C5-Fc elicits high, sustained serum antibody concentrations, prolonged neutralizing activity and long-term protection against multiple SARS-CoV-2 variants in both mice and common marmosets. These results align with earlier AAV-vectored antibody studies (including HIV and other viral pathogens) and extend them by demonstrating efficacy against contemporary SARS-CoV-2 variants; while significant translational challenges remain, the present findings support further preclinical optimization and safety evaluation as prerequisites for potential clinical development.

## Data Availability

The original contributions presented in the study are included in the article/supplementary material. Further inquiries can be directed to the corresponding author.
